# Comparative efficacy of radiofrequency denervation in chronic low back pain: A systematic review and network meta-analysis

**DOI:** 10.3389/fsurg.2022.899538

**Published:** 2022-08-05

**Authors:** Han Li, Junyan An, Jun Zhang, Weijian Kong, Zhihe Yun, Tong Yu, Xinyu Nie, Qinyi Liu

**Affiliations:** ^1^ Department of Respiratory and Critical Care Medicine, The Second Hospital of Jilin University, Changchun, China; ^2^ Department of Orthopedics, The Second Hospital of Jilin University, Changchun, China

**Keywords:** zygapophyseal joint, low back pain, radiofrequency therapy, denervation, network meta-analysis

## Abstract

**Background:**

Facet joint pain is a common cause of chronic low back pain (CLBP). Radiofrequency (RF) denervation is an effective treatment option.

**Purpose:**

A systematic review and network meta-analysis (NMA) was performed to evaluate and compare the efficacy and effectiveness of different RF denervation treatments in managing facet joint-derived CLBP.

**Methods:**

The Cochrane Library, Embase, PubMed, and China Biology Medicine were searched to identify eligible randomized controlled trials (RCTs) from January 1966 through December 2021. Interventions included conventional radiofrequency denervation (CRF), pulsed radiofrequency denervation (PRF), pulsed radiofrequency treatment of the dorsal root ganglia (PRF-DRG), radiofrequency facet capsule denervation (RF-FC), and radiofrequency ablation under endoscopic guidance (ERFA). The outcome was the mean change in visual analog scale (VAS) score from baseline. A random-effects NMA was used to compare the pain relief effects of the interventions over the short term (≤6 months) and long term (12 months). The rank of effect estimation for each intervention was computed using the surface under the cumulative ranking curve.

**Results:**

A total of 10 RCTs with 715 patients met the inclusion criteria. Moderate evidence indicated that CRF denervation had a greater effect on pain relief than sham control in the short term (standardized mean difference (SMD) −1.58, 95% confidence intervals (CI) −2.98 to −0.18) and the long term (SMD −4.90, 95% CI, −5.86 to −3.94). Fair evidence indicated that PRF denervation was more effective than sham control for pain over the long term (SMD −1.30, 95% CI, −2.17 to −0.43). Fair evidence showed that ERFA denervation was more effective for pain relief than sham control in the short term (SMD −3.07, 95% CI, −5.81 to −0.32) and the long term (SMD −4.00, 95% CI, −4.95 to −3.05). Fair evidence showed that RF-FC denervation was more effective for pain relief than sham control in the long term (SMD −1.11, 95% CI, −2.07 to −0.15). A fair level of evidence indicated that PRF-DRG denervation was more effective for pain relief than sham control in the short term (SMD −5.34, 95% CI, −8.30 to −2.39).

**Conclusion:**

RF is an effective option for patients diagnosed with facet joint-derived CLBP.

**Systematic Review Registration:** Identifier: CRD42022298238.

## Introduction

Low back pain is a worldwide health care problem with significant social and economic consequences. Most patients can be successfully treated in primary health care, but approximately 10%–15% have persistent pain that transforms into chronic low back pain (CLBP) (1). CLBP may be secondary to changes in the intervertebral discs, sacroiliac joints, and facet joints of the lumbar spine (2). Facet joint pain, which represents 10%–40% of CLBP, is characterized by a diffuse distribution between the L1-S1 segments (3). A 50% decrease in pain intensity after injection of local anesthetic into the medial branch can provide a definitive diagnosis of facet joint-derived CLBP (4).

Radiofrequency (RF) denervation, an invasive therapy for CLBP, is a technique that reduces spinal pain by modulating the neurotransmission of nociceptive stimuli. The transmission of nociceptive impulses is blocked by applying an electric current to coagulate the sensory nerves, which deactivates the nerves (2). A recent systematic review supported the superiority of conventional radiofrequency (CRF) over sham controls and other treatments in terms of short-term (≤6 months) and long-term (>6 months) improvement (5). However, there has been no systematic review of the effectiveness evaluation of other emerging RF denervation treatments, such as pulsed RF denervation, RF facet capsule denervation, and RF ablation under endoscopic guidance. The current systematic review was performed to evaluate the efficacy and effectiveness of different RF denervation treatments in managing facet joint-derived CLBP, and the literature search was updated through December 2021.

## Methods

A systematic review and network meta-analysis (NMA) was performed according to Preferred Reporting Items for Systematic Reviews and Meta-Analyses (PRISMA) to evaluate and compare the efficacy and effectiveness of different RF denervation treatments in managing CLBP of facet joint origin.

### Search strategies

#### Literature search

A comprehensive literature search was conducted to include randomized control trials (RCTs) published from all countries. Two experienced researchers (Han Li and Junyan An) comprehensively searched the Cochrane Library, Embase, PubMed, and China Biology Medicine independently by combining the following keywords: (“zygapophyseal joint” or “facet joint” or “facet osteoarthritis” or “back pain” or “backache” or “vertebrogenic pain” or “lumbago” or “lumbar pain”) to identify related articles published in English or Chinese between January 1966 and December 2021. Searches were also conducted for previous systematic reviews and cross-references. A detailed search strategy is provided in the **Supplementary material**. The third researcher (Jun Zhang) resolved the disagreements.

#### Inclusion and exclusion criteria

The inclusion criteria were as follows: (1) studies: RCTs; (2) participants: adult patients with low back pain lasting more than one month at the time of admission who were diagnosed with facet joint syndrome by a single or double diagnostic block and received at least three months of follow-up; (3) interventions: CRF, pulsed radiofrequency denervation (PRF), pulsed radiofrequency treatment of the dorsal root ganglia (PRF-DRG), radiofrequency facet capsule denervation (RF-FC), and radiofrequency ablation under endoscopic guidance (ERFA); and (4) outcome measures: the primary outcome measure was pain relief, and the outcome indicator was the visual analog scale (VAS). VAS represented 0 with no pain and 10 with the worst pain imaginable. The outcomes of 6 months or fewer of management were considered short-term, and 12 months was considered long-term. For RCTs with more than one follow-up, each follow-up period for VAS was categorized as short-term (≤6 months) and long-term (12 months) in this NMA.

The exclusion criteria were as follows: (1) studies in which the subject had an acute cause of low back pain, including fracture, osteoporosis, and malignancy; (2) letters, conference abstracts, and commentaries; (3) different studies recruiting the same participants; and (4) studies from which we could not extract the essential data.

### Data extraction

Two independent researchers (Han Li and Junyan An) extracted data from the included articles in a standardized data collection form, and a third researcher (Weijian Kong) validated the data extraction. Extracted data included (1) basic information: first author, region of study, study scale, study characteristic, and follow-up; (2) participants: gender distribution, age distribution, number of chronic low back pain patients, and duration of symptoms at enrollment; (3) therapy: protocol and target of interventions; and (4) outcomes: pain relief (the change in mean score on the VAS from baseline).

### Quality assessment

RCTs meeting the inclusion criteria were evaluated with version 2 of the Cochrane tool for assessing risk of bias in randomized trial (RoB2, revised version 2019) and Interventional Pain Management Techniques Quality Appraisal of Reliability and Risk of Bias Assessment (IPM-QRB) criteria (6). RCTs with scores of 32–48 and 16–31 were assessed as high in quality and moderate in quality, respectively. RCTs with scores under 16 were considered low in quality and were excluded from the NMA. The methodological quality of the RCTs was assessed independently by two researchers (Han Li and Junyan An). When discrepancies appeared, a third researcher (Zhihe Yun) was involved to resolve the conflict.

The qualitative analysis of the evidence was performed based on best-evidence synthesis, modified, and collated using multiple criteria, as shown in the **Supplementary material** (7). The qualitative analysis was conducted using five levels of evidence ranging from strong to opinion- or consensus-based. Two independent researchers (Han Li and Junyan An) analyzed the evidence in a standardized manner. Any disagreements between researchers were resolved by a third researcher (Qinyi Liu), and consensus was attained.

### Statistical analysis

The change in the mean VAS score from baseline extracted as the primary outcome was reported as the standardized mean difference (SMD) with 95% confidence interval (CI). The Higgins I^2^ statistic was calculated and the Cochran Q test was conducted to evaluate heterogeneity. Random-effects NMA was performed using STATA (version 14.0; StataCorp) (8–10). Indirect and mixed comparisons of NMA were conducted using the mvmeta and network commands of STATA. Heterogeneity was evaluated using the restricted maximum likelihood method and assuming a common heterogeneity variable (tau value) for all comparisons. Global inconsistencies, representing the plausibility of inconsistency in the entire network, were assessed with a design-by-treatment model. Local inconsistencies, representing the plausibility of inconsistency in the loop network, were estimated by a node-splitting method. The rank of effect estimation for each intervention was computed using the surface under the cumulative ranking curve (SUCRA). Publication bias was evaluated by funnel plots.

## Results

### Search results

**Figure 1:**
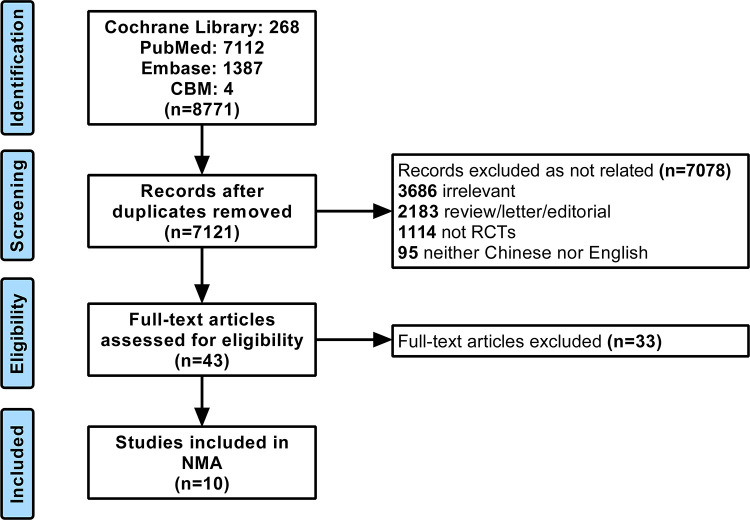
Flow chart of the literature search and selection of studies.

Our search yielded 8,771 records according to the predefined search strategy, of which 1,650 records were duplicates. A total of 7,078 studies were excluded after browsing the abstract. The full texts of 43 RCTs were retrieved for a detailed evaluation. Finally, we identified 10 RCTs for the NMA. The PRISMA flowchart is shown in Figure 1.

### Study characteristics

The study sample size for 10 RCTs ranged from 30 to 150 patients (11–20). Overall, 715 patients were included in the final analysis, of which 319 patients received CRF, 76 patients received PRF, 50 patients received ERFA, 50 patients received PRF-DRG, 40 patients received RF-FC, and 180 patients received a sham control of CRF after local anesthetic injection (CRF-sham). All RCTs induced a CRF group, which performed radiofrequency denervation of the medial branch of the posterior primary ramus at 80°C–85°C for 60–90 s. The intervention group for the three RCTs was PRF treatment (two Hertz at 42°C for 120–240 s) (15, 18, 19). Two RCTs compared ERFA with CRF (12, 14). ERFA involves endoscopic dissection of the dorsal medial branch and ablation with a radiofrequency cutting head. A separate RCT evaluated the efficacy of PRF-DRG, a percutaneous pulsed radiofrequency treatment of the dorsal root ganglia (13). Of the included RCTs, six reported both short-term (≤6 months) and long-term (12 months) outcomes, and four reported only short-term outcomes. The **Supplementary material** summarizes the details and the risk of bias of the included RCTs.

### Efficacy of interventions measured in NMA

**Figure 2:**
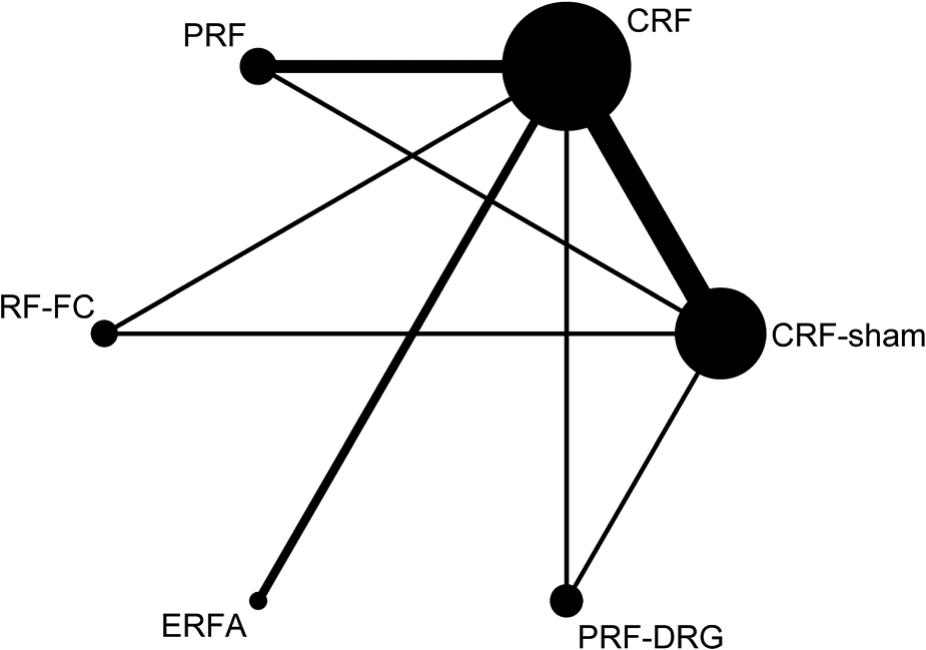
Network of eligible comparisons for the management of CLBP. The line indicates direct comparison of interventions, and the thickness of the line corresponds to the number of patients in the comparison. The size of the node corresponds to the number of studies that involve the intervention.

**Figure 3:**
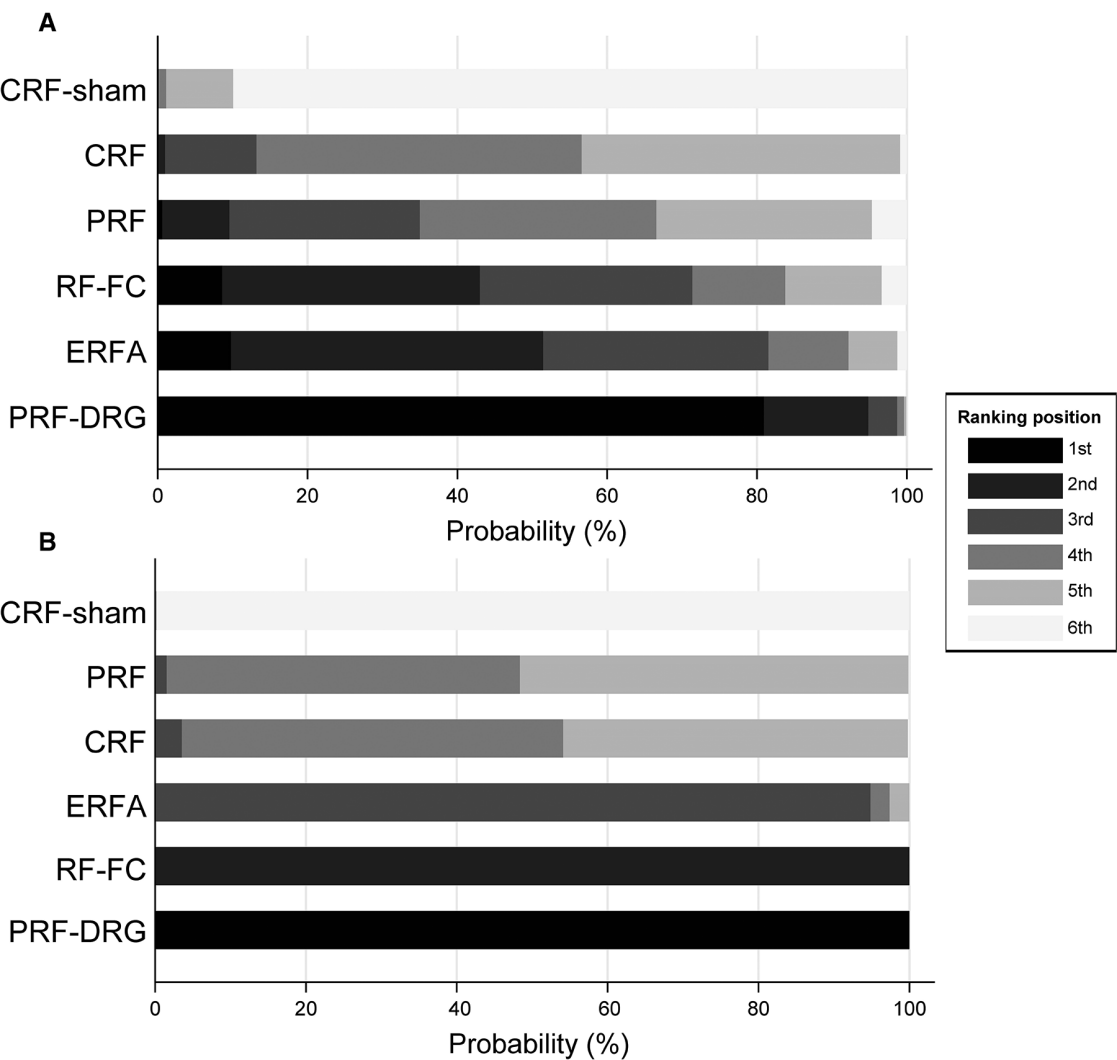
Treatment rank probabilities for pain relief for short-term (**A**) and long-term (**B**) follow-up. The order of the interventions on the vertical coordinate is based on the efficacy from lowest to highest. The horizontal coordinate is the probability of ranking 1st–6th.

**Figure 4:**
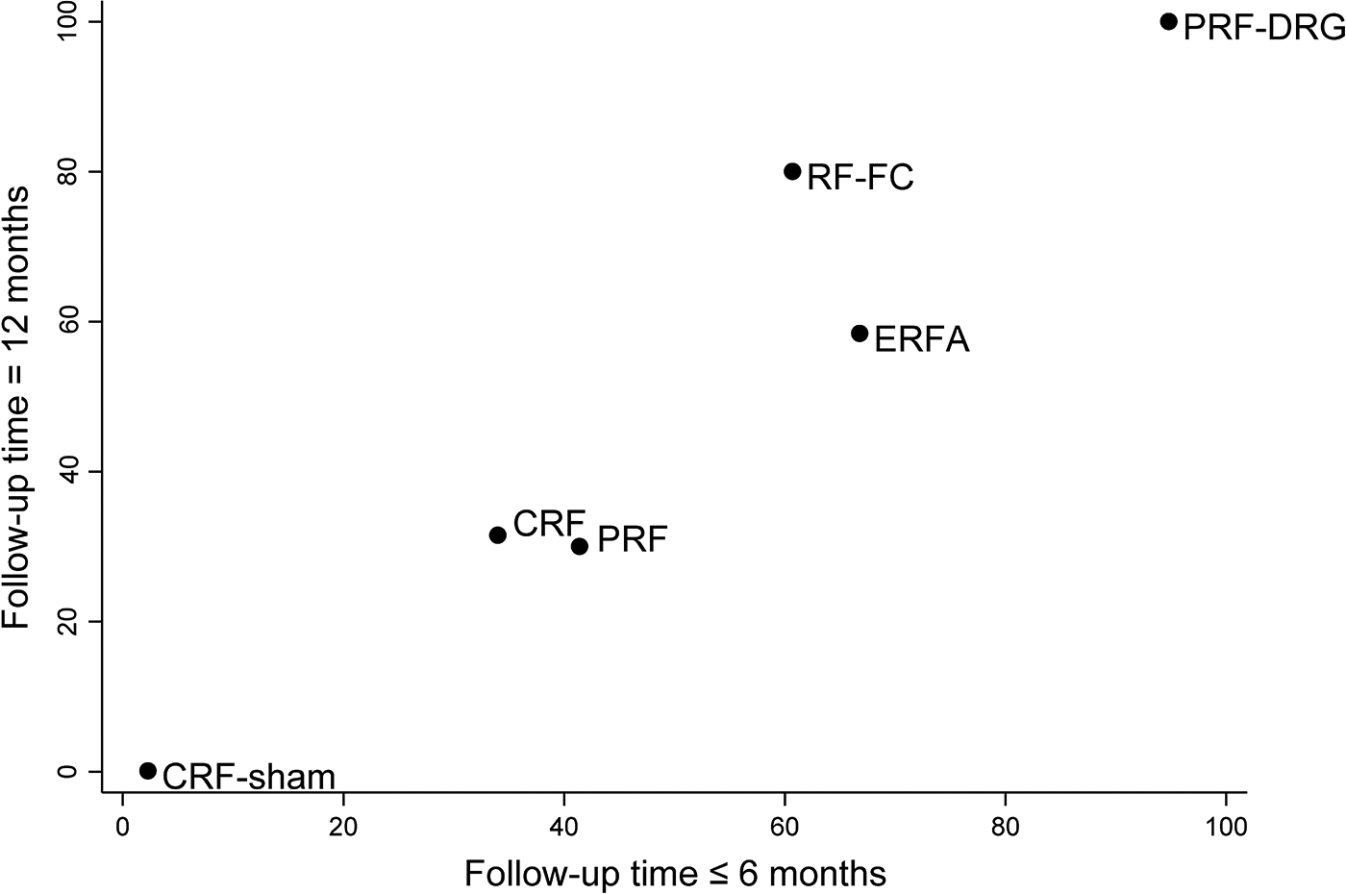
Scatter plot of the SUCRA for each intervention. The horizontal and vertical coordinates are the area under the SUCRA for each intervention at short-term and long-term follow-up, respectively. Higher values indicate higher efficacy ranking.

Figure 2 shows the network of eligible comparisons for the RF denervation options for CLBP. There was no evidence of heterogeneity or inconsistency in the NMA for short-term outcomes, but there was significant inconsistency in the NMA for long-term outcomes. Therefore, we fit an inconsistency model for long-term outcomes. Figure 3 shows the treatment rank probabilities for pain relief for short-term and long-term follow-up. Figure 4 shows a scatter plot based on the area under the SUCRA for each intervention. The results of the short-term and long-term effects of each intervention compared with other interventions are shown in Table 1. Moderate evidence indicated that CRF denervation had a greater effect on pain relief than sham control in the short term (SMD −1.58, 95% CI, −2.98 to −0.18) and the long term (SMD −4.90, 95% CI, −5.86 to −3.94). Fair evidence indicated that PRF denervation was more effective than sham control for pain over the long term (SMD −1.30, 95% CI, −2.17 to −0.43). Fair evidence showed that ERFA denervation was more effective for pain relief than sham control in the short term (SMD −3.07, 95% CI, −5.81 to −0.32) and the long term (SMD −4.00, 95% CI, −4.95 to −3.05). Fair evidence showed that RF-FC denervation was more effective for pain relief than sham control in the long term (SMD −1.11, 95% CI, −2.07 to −0.15). A fair level of evidence indicated that PRF-DRG denervation was more effective for pain relief than sham control in the short term (SMD −5.34, 95% CI, −8.30 to −2.39).

**Table 1:** League table for NMA of change in mean VAS score from baseline.


**Short-term (≤ 6 months)**					
**CRF-sham**	**−1.58** (**−2.98, −0.18)**	**−**1.82 (**−**4.02, 0.37)	−2.85 (−5.81, 0.11)	**−3.07** (**−5.81, −0.32)**	**−5.34** (**−8.30, −2.39)**
**4.90** (**3.94, 5.86)**	**CRF**	−0.24 (−2.16, 1.67)	−1.27 (−4.23, 1.69)	−1.49 (−3.85, 0.87)	−**3.76** (−**6.72,** −**0.81)**
**1.30** (**0.43, 2.17)**	**1.10** (**0.20, 2.00)**	**PRF**	−1.03 (−4.50, 2.44)	−1.24 (−4.28, 1.80)	−**3.52** (−**6.99,** −**0.06)**
**1.11** (**0.15, 2.07)**	**5.10** (**4.14, 6.06)**	**4.00** (**3.07, 4.93)**	**RF-FC**	−0.21 (−4.00, 3.57)	−2.49 (−6.56, 1.57)
**4.00** (**3.05, 4.95)**	**2.00** (**1.62, 2.37)**	0.90 (−0.07, 1.87)	−**3.10** (−**4.13,** −**2.07)**	**ERFA**	−2.28 (−6.06, 1.50)
0.20 (−0.70, 1.10)	**7.10** (**6.15, 8.05)**	**6.00** (**5.08, 6.92)**	**2.00** (**1.56, 2.44)**	**5.10** (**4.08, 6.12)**	**PRF-DRG**
**Long-term (12 months)**					

Short-term (upper right portion) and long-term (lower left portion) NMA results are presented for the mean change in VAS (from baseline) outcomes. Comparison should be made from left to right. Effect estimation is presented in standardized mean difference (SMD) with 95% confidence interval (CI), and the results are located between the column-defining intervention and row-defining intervention. For short-term (upper right portion) outcomes, an SMD less than 0 favors column-defining treatment. For long-term (lower left portion) outcomes, an SMD greater than 0 favors row-defining treatment. As a greater mean change in VAS score from baseline reflects greater pain relief, an increase in the absolute value of the SMD suggests better intervention for managing chronic low back pain. Significant results are marked in bold. CRF, conventional radiofrequency denervation; PRF, pulsed radiofrequency denervation; PRF-DRG, pulsed radiofrequency treatment of the dorsal root ganglia; RF-FC, radiofrequency facet capsule denervation; ERFA, radiofrequency ablation under endoscopic guidance; CRF-sham, a sham control of CRF.

## Discussion

### Summary of main results

The purpose of this systematic review and network meta-analysis (NMA) was to evaluate the effectiveness of different radiofrequency (RF) denervation procedures for the management of chronic low back pain (CLBP) based on information provided by randomized controlled trials (RCTs). We included 10 RCTs with five interventions: conventional radiofrequency denervation (CRF), pulsed radiofrequency denervation (PRF), pulsed radiofrequency treatment of the dorsal root ganglia (PRF-DRG), radiofrequency facet capsule denervation (RF-FC), and radiofrequency ablation under endoscopic guidance (ERFA). Of these, 60% were considered to have a low risk of bias. The reviewed RCTs provided evidence of fair to moderate quality, suggesting that CRF, ERFA, and PRF-DRG denervation could offer greater pain relief for short-term follow-up than sham surgery, whereas PRF, CRF, ERFA, and RF-FC could offer greater pain relief for long-term follow-up.

### Agreements and disagreements with other studies or reviews

In 2021, Janapala et al. (5) published a systematic review on CRF in CLBP that included a dual-arm meta-analysis of pain relief with six RCTs and a single-arm meta-analysis of pain relief with 10 RCTs. They concluded that moderate evidence could support CRF procedures over sham control and other treatments for both short-term (≤6 months) and long-term (>6 months) improvement. This finding was consistent with our results suggesting that CRF denervation was more effective than sham control in managing CLBP of facet joint origin. Although CRF is an effective therapy for pain relief, several adverse effects, including localized pain at the lesion site and neuritic pain, have been reported (21). Unfortunately, all previously published systematic reviews noted that adverse effects were not sufficiently reported. PRF uses less energy and lower temperature than CRF, which avoids neuronal tissue damage (22). In 2019, Contreras Lopez et al. (3) published a systematic review on PRF in CLBP including three RCTs. They indicated that PRF was less effective than CRF in relieving pain and restoring function and recommended the use of CRF with a high safety profile after conventional treatment. The results of our NMA showed that there was no significant difference in pain relief in the short-term follow-up between CRF and PRF (SMD −0.24, 95% CI, −2.16 to 1.67). The results of long-term follow-up showed that CRF was less effective than PRF (SMD 1.10, 95% CI, 0.20 to 2.00). However, when compared with sham controls, CRF (SMD −4.90, 95% CI, −5.86 to −3.94) appeared to produce more significant pain relief than PRF (SMD −1.30, 95% CI, −2.17 to −0.43). In conclusion, our systematic review could not lead to any conclusions regarding the comparative efficacy of CRF and PRF.

### Limitations of the systematic review

First, the low total number of patients included in the NMA resulted in a low overall completeness of the evidence. From a clinical point of view, the overall low number of patients is understandable due to the potential damage to patients from x-ray exposure with this invasive technique. However, this methodological shortcoming inevitably leads to a lower quality of evidence.

Second, while PRF-DRG denervation showed favorable outcomes in the short term, the result was measured from a single RCT (13), reflecting the value of further RCTs to substantiate this finding.

Third, of the 10 RCTs included in the NMA, only four reported indicators of pain and disorder-specific disability. In this systematic review, we did not include “disorder-specific disability”, “treatment-related costs”, or “ability to work” as required criteria. This was partly because these indicators are not always relevant in patients with CLBP and partly due to the limitations of the trial design of the included RCTs.

Fourth, we did not draw definitive conclusions about the risks of RF denervation due to the small size of the RCTs included in the NMA and the lack of assessment of adverse events.

Fifth, the follow-up time varied from three months to three years. Three RCTs had a follow-up of less than one year, resulting in missing long-term outcomes. Although two RCTs were performed with up to three years of follow-up, no data were extracted due to the inevitably large proportion of missed visits at the two- and three-year follow-ups. Nevertheless, longer follow-up periods are necessary to demonstrate the effectiveness of RF denervation.

Sixth, the lack of RCTs with low bias was a major limitation of this systematic review, although it is encountered in many other systematic reviews. In addition, in most of the RCTs included in the NMA, it was not clearly reported whether cointerventions or similar interventions were avoided. Methodologically sound RCTs with adequate sample sizes performed to assess the effectiveness of RF denervation are still rare.

Finally, we attempted to minimize the potential of publication bias through an extensive database search (through December 2021). Although the funnel plot showed no significant publication bias for the included RCTs, it was not possible to assess the impact of potential publication bias on the results.

## Conclusion

In this systematic review, we analyzed current RCTs regarding different RF treatments in managing CLBP of facet joint origin. The evidence suggested that CRF, ERFA, and PRF-DRG denervation could offer greater pain relief for short-term follow-up than sham surgery, whereas PRF, CRF, ERFA, and RF-FC could offer greater pain relief for long-term follow-up. We concluded that RF is an effective option for patients diagnosed with facet joint-derived CLBP. However, high-quality RCTs with larger patient samples and long-term follow-up results are needed.

## Data Availability

The original contributions presented in the study are included in the article/**Suplementary Material**, further inquiries can be directed to the corresponding author/s.
