# Supplementary material for: Comparative efficacy of radiofrequency denervation in chronic low back pain: A systematic review and network meta-analysis
Source: Front Surg. 2022 Aug 5;9:899538. doi: 10.3389/fsurg.2022.899538 (PMC9388860; doi:10.3389/fsurg.2022.899538)
Supplement: Supplementary file 1 [file Table_Supplementary_updated.docx]

Comparative Efficacy of Radiofrequency Denervation in Chronic Low Back Pain: A Systematic Review and Network Meta-Analysis

Supplementary Appendix

A. Search terms for PubMed

1. Zygapophyseal Joint[Title/Abstract]

2. zygapophyseal joint*[Title/Abstract]

3. zygapophysial joint*[Title/Abstract]

4. facet joint[Title/Abstract]

5. facet joint*[Title/Abstract]

6. facet joint syndrome[Title/Abstract]

7. facet joint osteoarthritis[Title/Abstract]

8. facet osteoarthritis[Title/Abstract]

9. facet joint synovial cysts[Title/Abstract]

10. "Zygapophyseal Joint"[Mesh]

11. Back Pain[Title/Abstract]

12. Backache[Title/Abstract]

13. Back ache[Title/Abstract]

14. Vertebrogenic Pain Syndrome[Title/Abstract]

15. "Back Pain"[Mesh]

16. Low Back Pain[Title/Abstract]

17. lumbago[Title/Abstract]

18. lumbar pain[Title/Abstract]

19. "Low Back Pain"[Mesh]

20. OR/1-19

21. "Randomized Controlled Trial" [Publication Type]

22. randomized controlled trial[Title/Abstract]

23. controlled clinical trial[Title/Abstract]

24. randomized controlled trials[Title/Abstract]

25. random allocation[Title/Abstract]

26. double-blind method[Title/Abstract]

27. single-blind method[Title/Abstract]

28. clinical trial[Title/Abstract]

29. clinical trials[Title/Abstract]

30. OR/21-19

31. 20 AND 30

B. Table 1. Qualitative modified approach to grading of evidence of therapeutic effectiveness studies

| Level I | Strong | Evidence obtained from multiple relevant high-quality randomized controlled trials |
| --- | --- | --- |
| Level Ⅱ | Moderate | Evidence obtained from at least one relevant high-quality randomized controlled trial or multiple relevant moderate or low-quality randomized controlled trials |
| Level Ⅲ | Fair | Evidence obtained from at least one relevant moderate or low-quality randomized trial or Evidence obtained from at least one relevant high-quality non-randomized trial or observational study with multiple moderate or low-quality observational studies |
| Level Ⅳ | Limited | Evidence obtained from multiple moderate or low-quality relevant observational studies |
| Level Ⅴ | Consensus based | Opinion or consensus of large group of clinicians and/or scientists |

C. Table 2. Characteristics of included RCTs

| First author  (year) | Region | Scale | Study characteristic | Duration of symptoms at enrollment | Final follow-up time | Loss to follow-up | Female |
| --- | --- | --- | --- | --- | --- | --- | --- |
| van Kleef, M.  (1999)[1] | The Netherlands | Single center | RA; DB | >12 months | 12 months | 0% | 65% |
| Leclaire, R.  (2001)[2] | Canada | Single center | RA; DB | >3 months | 3 months | 5.6% at 3months | 64% |
| Tekin, Idil  (2007)[3] | Turkey | Single center | RA; DB | >6 months | 12 months | 0% | 57% |
| Kroll, Henry R.  (2008)[4] | USA | Single center | RA; DB | >1 month | 3 months | 48% vs. 48% | 54% |
| Nath, Sherdil.  (2008)[5] | Sweden | Single center | RA; DB | >24 months | 6 months | 0% | 63% |
| Moussa, W. M.  (2016)[6] | Egypt | Single center | RA; DB | >24 months | 36 months | 8% at 1 year | 73% |
| Çetin, Abdurrahman.  (2018)[7] | Turkey | Single center | RA; DB | mean 13.5 months | 24 months | 0% | 61% |
| Song, K.  (2019)[8] | China | Single center | RA | >3 months | 24 months | 0% | 40% |
| Moussa, W. M.  (2020)[9] | Egypt | Single center | RA; DB | >12 months | 36 months | 5% at 1 year | 65% |
| Xue, Y.  (2020)[10] | China | Single center | RA | >3 months | 12 months | 0% | 46% |

(continued)

| First author  (year) | Intervention A | N  (A) | Age  (A, mean±SD) | Intervention B | N  (B) | Age  (B, mean±SD) |
| --- | --- | --- | --- | --- | --- | --- |
| van Kleef, M.  (1999) | CRF (80 °C for 60 seconds) | 15 | 46.5±7.4 | - | - | - |
| Leclaire, R.  (2001) | CRF (85 °C for 90 seconds) | 36 | 46.7±9.3 | - | - | - |
| Tekin, Idil.  (2007) | CRF (80 °C for 90 seconds) | 20 | 57.9±9.3 | PRF: pulsed RF neurotomy (two Hertz at 42 °C for 240 seconds) | 20 | 59.6±7.7 |
| Kroll, Henry R.  (2008) | CRF (80 °C for 75 seconds) | 13 | 59.5±11.6 | PRF: pulsed RF neurotomy (two Hertz at 42 °C for 120 seconds) | 13 | 57.0±8.4 |
| Nath, Sherdil.  (2008) | CRF (85 °C for 60 seconds) | 20 | 56 | - | - | 53 |
| Moussa, W. M.  (2016) | CRF (85 °C for 90 seconds) | 40 | 56.5 | RF-FC: conventional RF coagulation of the facet joint capsule (85 °C for 90 seconds) | 40 | 58.1 |
| Çetin, Abdurrahman.  (2018) | CRF (80 °C for 90 seconds) | 75 | 53.90±16.23 | PRF: pulsed RF neurotomy (two Hertz at 42 °C for 180 seconds) | 43 | 53.59±16.14 |
| Song, K.  (2019) | CRF (80 °C for 90 seconds) | 20 | - | ERFA: endoscopic neurotomy of lumbar medial branch | 20 | - |
| Moussa, W. M.  (2020) | CRF | 50 | 57.3 | PRF-DRG: percutaneous pulsed radiofrequency treatment of the dorsal root ganglia | 50 | 56.7 |
| Xue, Y.  (2020) | CRF (80 °C for 60 seconds once, and 90 °C for 80 seconds one more time) | 30 | 64.78±6.62 | ERFA: endoscopic neurotomy of lumbar medial branch | 30 | 65.73±7.62 |

(continued)

| First author  (year) | Control | N  (C) | Age  (C, mean±SD) | Target of the interventions |
| --- | --- | --- | --- | --- |
| van Kleef, M.  (1999) | CRF-sham | 16 | 41.4±7.5 | Medial branch of the posterior primary ramus |
| Leclaire, R.  (2001) | CRF-sham | 34 | 46.4±9.8 | Medial branch of the posterior primary ramus |
| Tekin, Idil.  (2007) | CRF-sham | 20 | 60.5±8.5 | Medial branch of the posterior primary ramus |
| Kroll, Henry R.  (2008) | - | - | - | Medial branch of the posterior primary ramus |
| Nath, Sherdil.  (2008) | CRF-sham | 20 | - | Medial branch of the posterior primary ramus |
| Moussa, W. M.  (2016) | CRF-sham | 40 | 55.9 | Medial branch of the posterior primary ramus (A) vs. facet joint capsule of L3-4, L4-5 and L5-S1 levels (B) |
| Çetin, Abdurrahman.  (2018) | - | - | - | Medial branch of the posterior primary ramus |
| Song, K.  (2019) | - | - | - | Medial branch of the posterior primary ramus |
| Moussa, W. M.  (2020) | CRF-sham | 50 | 56.9 | Medial branch of the posterior primary ramus (A) vs. dorsal root ganglia (B) |
| Xue, Y.  (2020) | - | - | - | Medial branch of the posterior primary ramus |

RA = randomized; DB = double-blind; RF = radiofrequency; CRF = conventional radiofrequency denervation; PRF = pulsed radiofrequency denervation; PRF-DRG = pulsed radiofrequency treatment of the dorsal root ganglia; RF-FC = radiofrequency facet capsule denervation; ERFA = radiofrequency ablation under endoscopic guidance; CRF-sham = a sham control of CRF; VAS = visual analog scale; RMQ = Roland-Morris questionnaire; ODI = Oswestry disability index.

D. Methodologic quality assessment of RCTs utilizing version 2 of the Cochrane tool for assessing risk of bias in randomized trial (RoB2, revised version 2019)


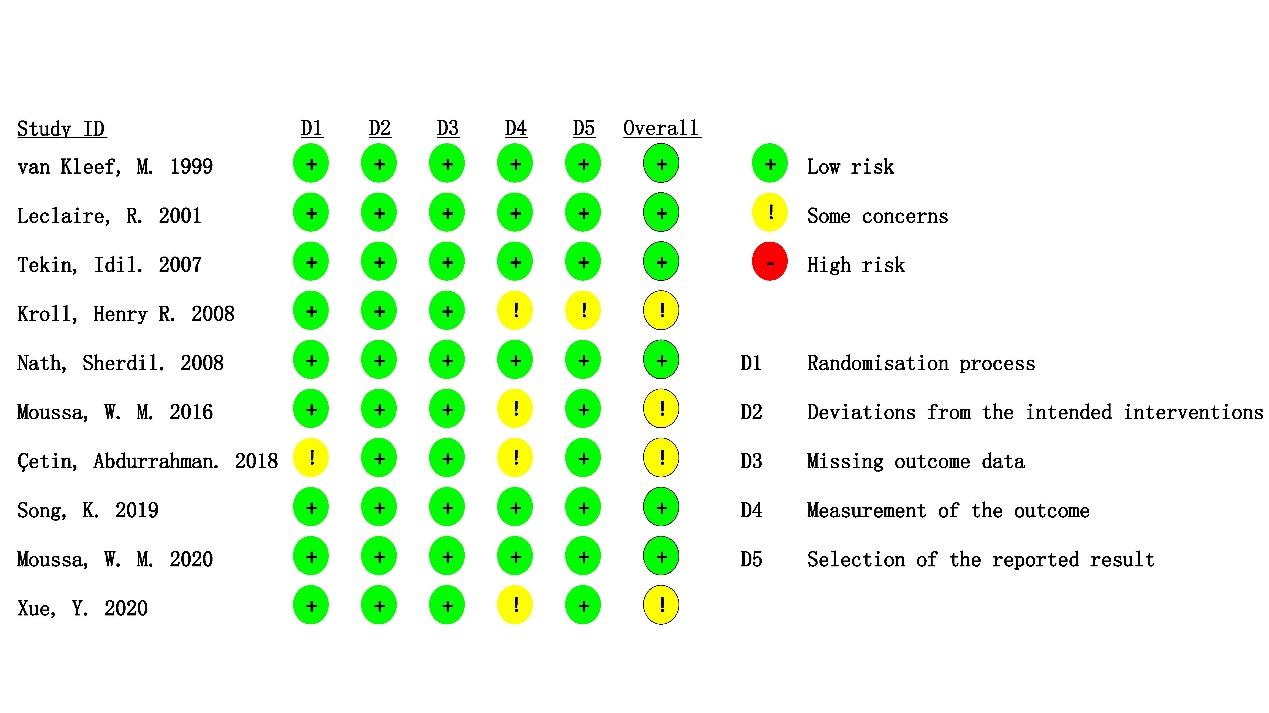


E. Table 3. Methodologic quality assessment of RCTs utilizing IPM – QRB criteria

|  | Study ID | van Kleef, M. 1999 | Leclaire, R. 2001 | Tekin, Idil. 2007 | Kroll, Henry R. 2008 | Nath, Sherdil. 2008 |
| --- | --- | --- | --- | --- | --- | --- |
|  | PMID | 10515020 | 11458140 | 31524141 | 19041042 | 18496338 |
| I. | TRIAL DESIGN AND GUIDANCE REPORTING |  |  |  |  |  |
| 1 | CONSORT or SPIRIT | 2 | 2 | 2 | 2 | 3 |
| II. | DESIGN FACTORS |  |  |  |  |  |
| 2 | Type and Design of Trial | 3 | 3 | 3 | 2 | 3 |
| 3 | Setting/Physician | 3 | 2 | 2 | 3 | 3 |
| 4 | Imaging | 3 | 3 | 3 | 3 | 3 |
| 5 | Sample Size | 1 | 2 | 1 | 1 | 1 |
| 6 | Statistical Methodology | 1 | 1 | 1 | 1 | 1 |
| III. | PATIENT FACTORS |  |  |  |  |  |
| 7 | Inclusiveness of Population | 1 | 2 | 1 | 2 | 2 |
| 8 | Duration of Pain | 3 | 1 | 2 | 1 | 2 |
| 9 | Previous Treatments | 0 | 1 | 1 | 1 | 0 |
| 10 | Duration of Follow-up with Appropriate Interventions | 2 | 1 | 2 | 1 | 2 |
| IV. | OUTCOMES |  |  |  |  |  |
| 11 | Outcomes Assessment Criteria for Significant Improvement | 2 | 0 | 2 | 1 | 4 |
| 12 | Analysis of all Randomized Participants in the Groups | 2 | 2 | 2 | 2 | 2 |
| 13 | Description of Drop Out Rate | 2 | 2 | 2 | 0 | 2 |
| 14 | Similarity of Groups at Baseline for Important Prognostic Indicators | 2 | 2 | 2 | 2 | 2 |
| 15 | Role of Co-Interventions | 1 | 1 | 1 | 0 | 1 |
| V. | RANDOMIZATION |  |  |  |  |  |
| 16 | Method of Randomization | 2 | 2 | 2 | 2 | 2 |
| VI. | ALLOCATION CONCEALMENT |  |  |  |  |  |
| 17 | Concealed Treatment Allocation | 2 | 2 | 2 | 1 | 2 |
| VII. | BLINDING |  |  |  |  |  |
| 18 | Patient Blinding | 1 | 1 | 1 | 1 | 1 |
| 19 | Care Provider Blinding | 1 | 1 | 1 | 1 | 1 |
| 20 | Outcome Assessor Blinding | 0 | 1 | 0 | 0 | 0 |
| VIII. | CONFLICTS OF INTEREST |  |  |  |  |  |
| 21 | Funding and Sponsorship | 3 | 2 | 2 | 1 | 2 |
| 22 | Conflicts of Interest | 3 | 3 | 2 | 0 | 3 |
| TOTAL | | 40 | 37 | 37 | 28 | 42 |

(continued)

|  | Study ID | Moussa, W. M. 2016 | Çetin, Abdurrahman. 2018 | Song, K. 2019 | Moussa, W. M. 2020 | Xue, Y. 2020 |
| --- | --- | --- | --- | --- | --- | --- |
|  | PMID | 27618781 | 30595777 | 30790724 | 33045627 | 31900227 |
| I. | TRIAL DESIGN AND GUIDANCE REPORTING |  |  |  |  |  |
| 1 | CONSORT or SPIRIT | 2 | 2 | 2 | 2 | 2 |
| II. | DESIGN FACTORS |  |  |  |  |  |
| 2 | Type and Design of Trial | 3 | 2 | 2 | 3 | 2 |
| 3 | Setting/Physician | 1 | 2 | 1 | 1 | 2 |
| 4 | Imaging | 3 | 3 | 3 | 3 | 3 |
| 5 | Sample Size | 2 | 2 | 0 | 3 | 2 |
| 6 | Statistical Methodology | 1 | 1 | 1 | 1 | 1 |
| III. | PATIENT FACTORS |  |  |  |  |  |
| 7 | Inclusiveness of Population | 2 | 2 | 2 | 3 | 3 |
| 8 | Duration of Pain | 2 | 2 | 1 | 2 | 1 |
| 9 | Previous Treatments | 2 | 2 | 2 | 2 | 2 |
| 10 | Duration of Follow-up with Appropriate Interventions | 3 | 3 | 3 | 3 | 2 |
| IV. | OUTCOMES |  |  |  |  |  |
| 11 | Outcomes Assessment Criteria for Significant Improvement | 2 | 2 | 2 | 2 | 0 |
| 12 | Analysis of all Randomized Participants in the Groups | 1 | 2 | 0 | 2 | 2 |
| 13 | Description of Drop Out Rate | 2 | 2 | 0 | 2 | 2 |
| 14 | Similarity of Groups at Baseline for Important Prognostic Indicators | 2 | 2 | 2 | 1 | 1 |
| 15 | Role of Co-Interventions | 1 | 1 | 1 | 1 | 1 |
| V. | RANDOMIZATION |  |  |  |  |  |
| 16 | Method of Randomization | 2 | 0 | 0 | 2 | 2 |
| VI. | ALLOCATION CONCEALMENT |  |  |  |  |  |
| 17 | Concealed Treatment Allocation | 2 | 0 | 0 | 2 | 2 |
| VII. | BLINDING |  |  |  |  |  |
| 18 | Patient Blinding | 1 | 1 | 0 | 1 | 0 |
| 19 | Care Provider Blinding | 0 | 0 | 0 | 0 | 0 |
| 20 | Outcome Assessor Blinding | 1 | 1 | 1 | 1 | 0 |
| VIII. | CONFLICTS OF INTEREST |  |  |  |  |  |
| 21 | Funding and Sponsorship | 3 | 2 | 3 | 3 | 3 |
| 22 | Conflicts of Interest | 3 | 0 | 3 | 3 | 3 |
| TOTAL | | 41 | 34 | 29 | 43 | 36 |

Source: Manchikanti L, et al. Assessment of methodologic quality of randomized trials of interventional techniques: Development of an interventional pain management specific instrument. Pain Physician 2014; 17:E263-E290 (527).

F. Heterogeneity, inconsistency, and publication bias evaluation for efficacy (mean change in VAS from baseline)

1. Short-term outcomes

1.1 Global inconsistency

Test of consistency: chi2(4)=4.52, P=0.340

1.2 Side-splitting method


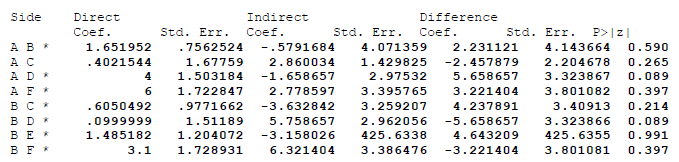


1.3 Loop-specific approach


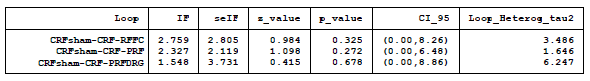


1.4 Comparison-adjusted funnel plot


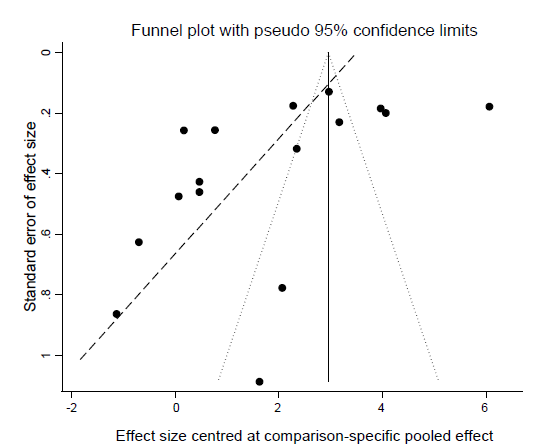


1.5 Forest plot


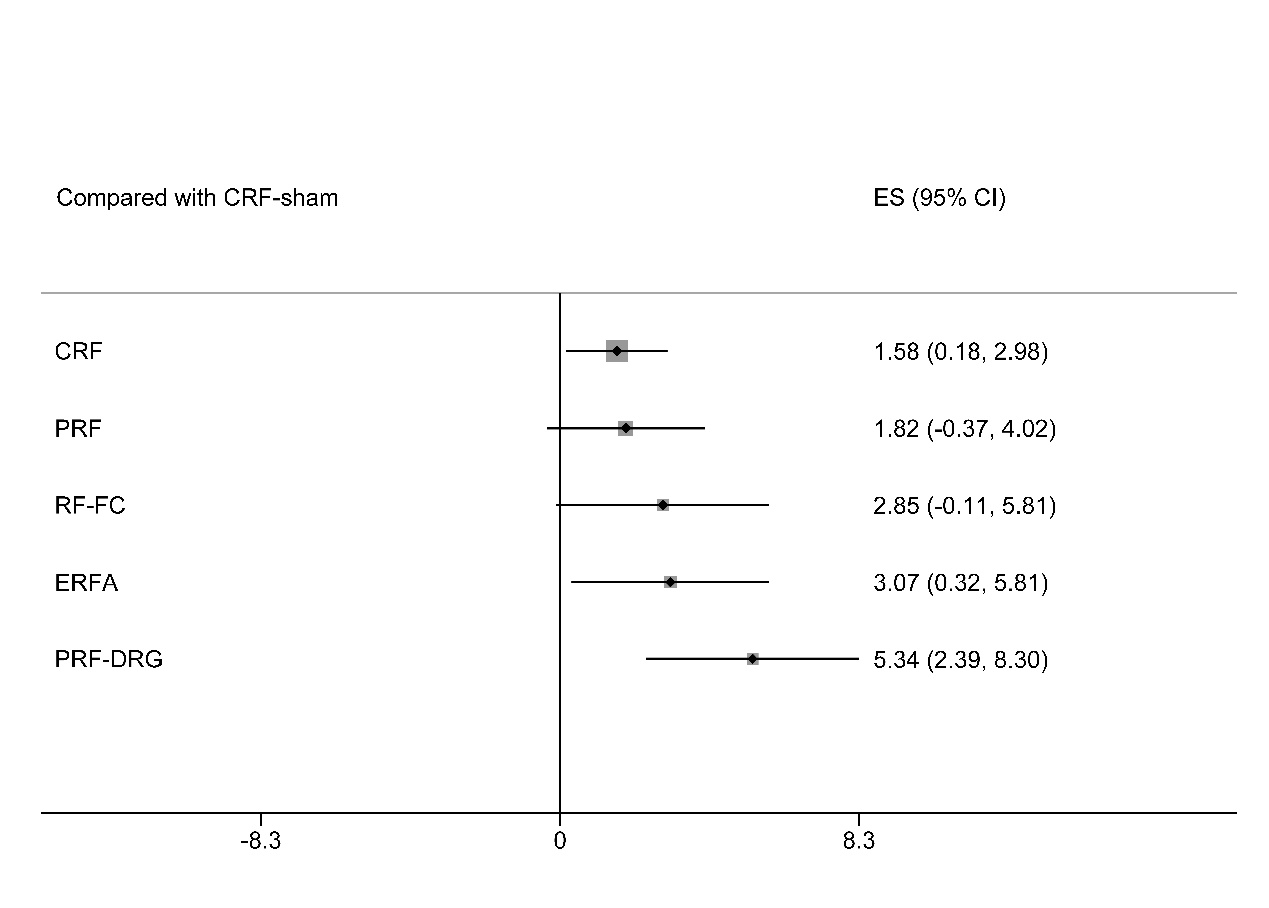


2. Long-term outcomes

2.1 Global inconsistency

Test of consistency: chi2(3)=110.23, P<0.001

2.2 Side-splitting method


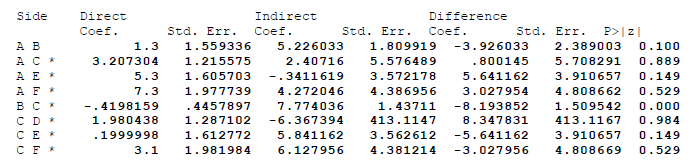


2.3 Comparison-adjusted funnel plot


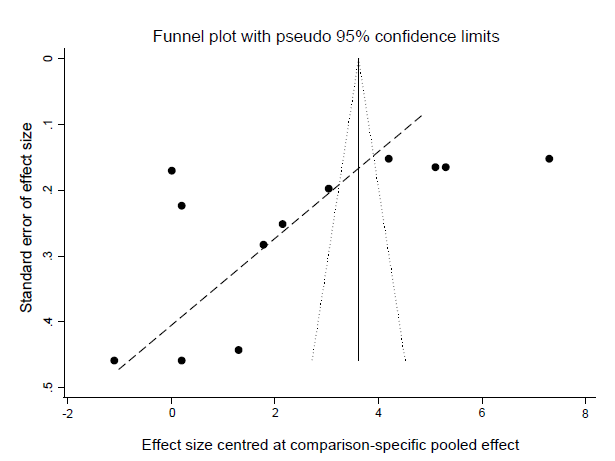


2.4 Forest plot


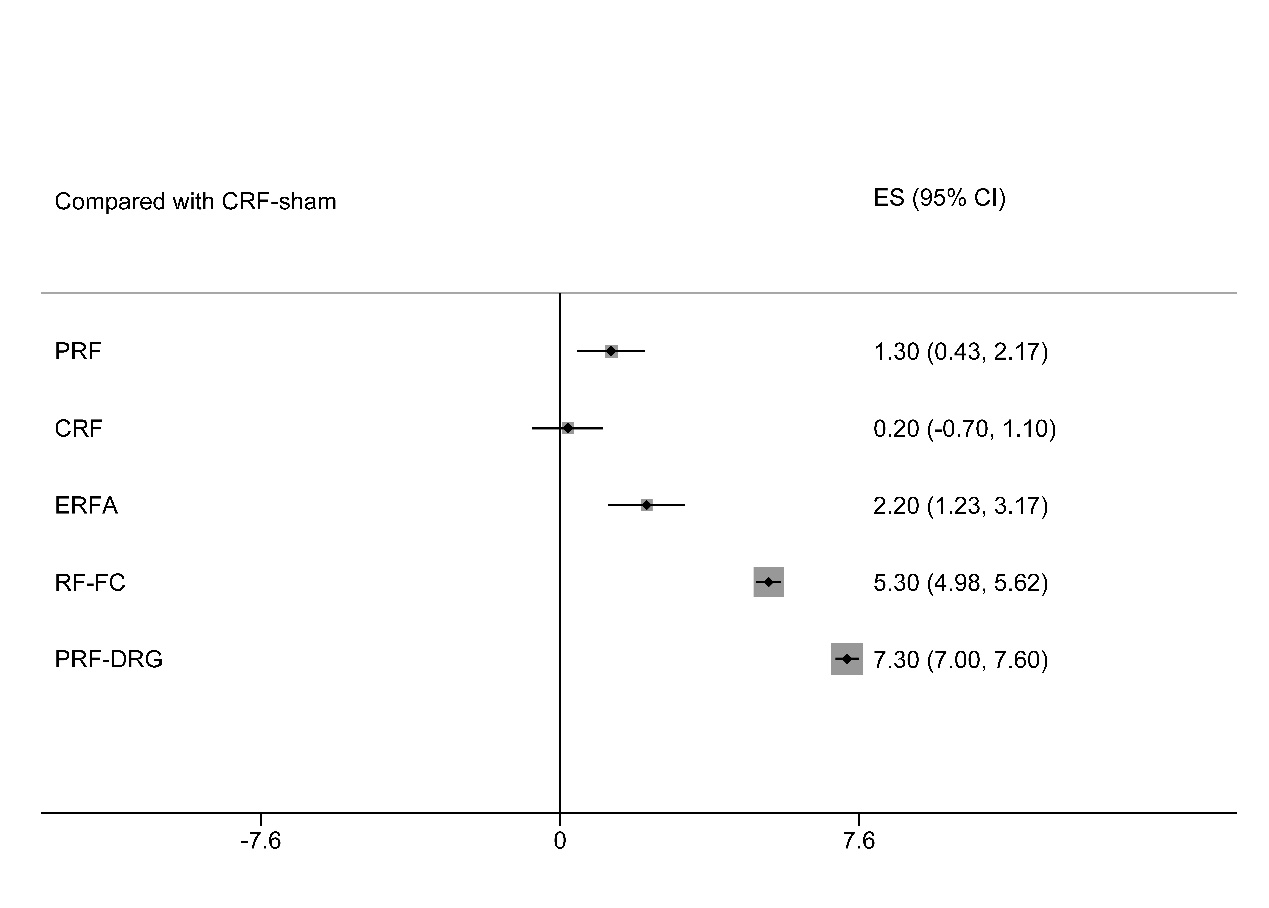


G. The surface under the cumulative ranking curve (SUCRA) for pain relief for short-term (A) and long-term (B) follow-up


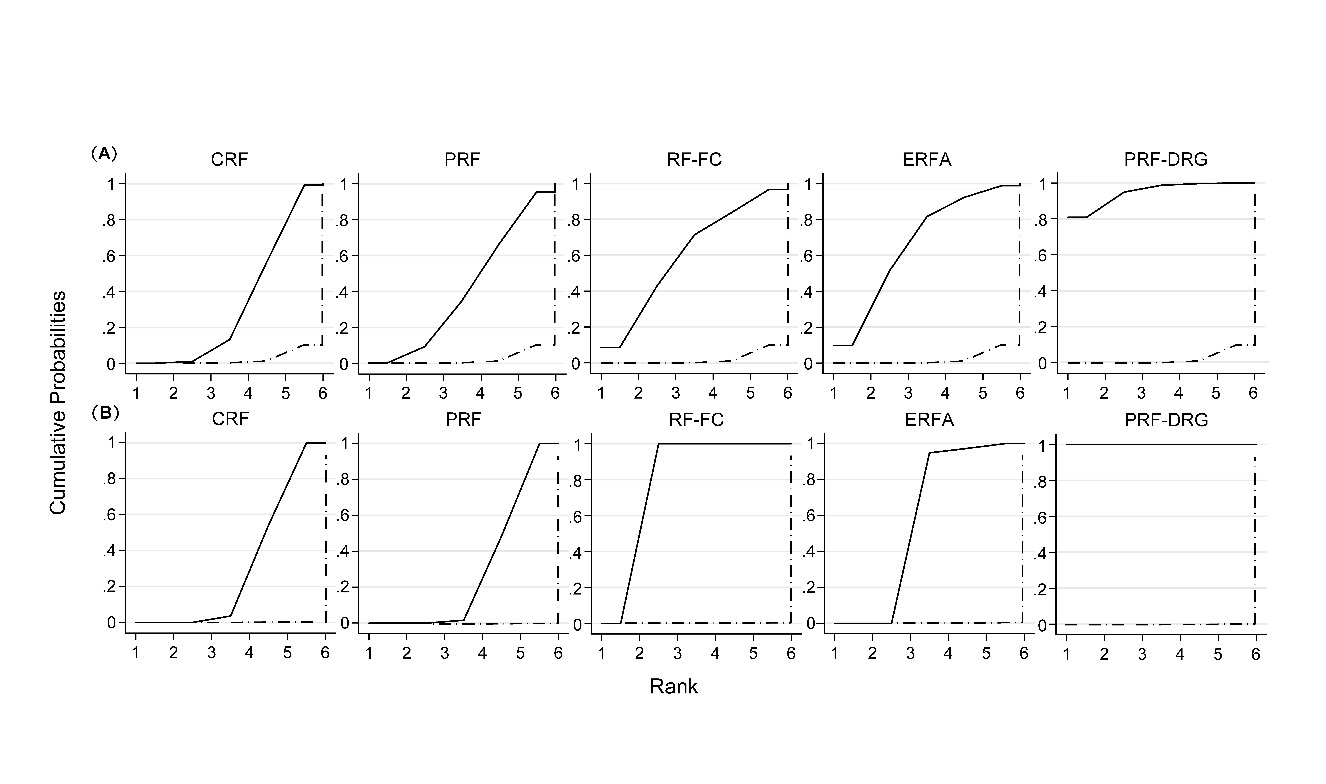


The surface under the cumulative ranking curve (SUCRA) was used to rank the effectiveness of each intervention and identify the best intervention. SUCRA values range from 0 to1, being the worst to the best. Dotted line: CRF-sham. CRF = conventional radiofrequency denervation; PRF = pulsed radiofrequency denervation; PRF-DRG = pulsed radiofrequency treatment of the dorsal root ganglia; RF-FC = radiofrequency facet capsule denervation; ERFA = radiofrequency ablation under endoscopic guidance; CRF-sham = a sham control of CRF.

REFERENCES

1. van Kleef M, Barendse GA, Kessels A, Voets HM, Weber WE, de Lange S. Randomized trial of radiofrequency lumbar facet denervation for chronic low back pain. Spine (Phila Pa 1976) 1999, 24(18):1937-1942.

2. Leclaire R, Fortin L, Lambert R, Bergeron YM, Rossignol M. Radiofrequency facet joint denervation in the treatment of low back pain: a placebo-controlled clinical trial to assess efficacy. Spine (Phila Pa 1976) 2001, 26(13).

3. Tekin I, Mirzai H, Ok G, Erbuyun K, Vatansever D. A comparison of conventional and pulsed radiofrequency denervation in the treatment of chronic facet joint pain. Clin J Pain 2007, 23(6):524-529.

4. Kroll HR, Kim D, Danic MJ, Sankey SS, Gariwala M, Brown M. A randomized, double-blind, prospective study comparing the efficacy of continuous versus pulsed radiofrequency in the treatment of lumbar facet syndrome. J Clin Anesth 2008, 20(7):534-537.

5. Nath S, Nath CA, Pettersson K. Percutaneous lumbar zygapophysial (Facet) joint neurotomy using radiofrequency current, in the management of chronic low back pain: a randomized double-blind trial. Spine (Phila Pa 1976) 2008, 33(12).

6. Moussa WM, Khedr W. Percutaneous radiofrequency facet capsule denervation as an alternative target in lumbar facet syndrome. Clin Neurol Neurosurg 2016, 150:96-104.

7. Çetin A, Yektaş A. Evaluation of the Short- and Long-Term Effectiveness of Pulsed Radiofrequency and Conventional Radiofrequency Performed for Medial Branch Block in Patients with Lumbar Facet Joint Pain. Pain research & management 2018, 2018:7492753.

8. Song K, Li Z, Shuang F, Yin X, Cao Z, Zhao H, Qin J, Li Z. Comparison of the Effectiveness of Radiofrequency Neurotomy and Endoscopic Neurotomy of Lumbar Medial Branch for Facetogenic Chronic Low Back Pain: A Randomized Controlled Trial. World Neurosurg 2019, 126:e109-e115.

9. Moussa WM, Khedr W, Elsawy M. Percutaneous pulsed radiofrequency treatment of dorsal root ganglion for treatment of lumbar facet syndrome. Clin Neurol Neurosurg 2020, 199:106253.

10. Xue Y, Ding T, Wang D, Zhao J, Yang H, Gu X, Feng D, Zhang Y, Liu H, Tang F et al. Endoscopic rhizotomy for chronic lumbar zygapophysial joint pain. J Orthop Surg Res 2020, 15(1):4.

**
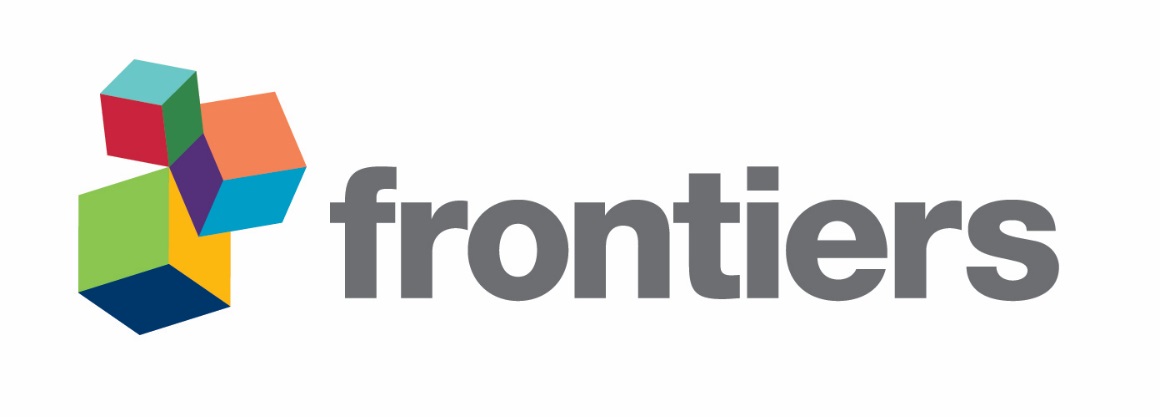
**
